# Reading skill modulates the effect of parafoveal distractors on foveal lexical decision in deaf students

**DOI:** 10.1371/journal.pone.0221891

**Published:** 2019-09-12

**Authors:** Jiayu Tao, Zhao Qin, Zhu Meng, Li Zhang, Lu Liu, Guoli Yan, Valerie Benson

**Affiliations:** 1 Academy of Psychology and Behavior, Tianjin Normal University, Tianjin, China; 2 Department of Psychology, Chengde Medical University, Chengde, Hebei, China; 3 School of Educational Science, Ludong University, Yantai, Shandong, China; 4 School of Psychology, University of Central Lancashire, Preston, Lancashire, United Kingdom; University of Florida, UNITED STATES

## Abstract

In low-level perceptual tasks and reading tasks, deaf individuals show a redistribution of spatial visual attention toward the parafoveal and peripheral visual fields. In the present study, the experiment adopted the modified flanker paradigm and utilized a lexical decision task to investigate how these unique visual skills may influence foveal lexical access in deaf individuals. It was predicted that irrelevant linguistic stimuli presented in parafoveal vision, during a lexical decision task, would produce a larger interference effect for deaf college student readers if the stimuli acted as distractors during the task. The results showed there was a larger interference effect in deaf college student readers compared to the interference effect observed in participants with typical levels of hearing. Furthermore, deaf college student readers with low-skilled reading levels showed a larger interference effect than those with high-skilled reading levels. The current study demonstrates that the redistribution of spatial visual attention toward the parafoveal visual regions in deaf students impacts foveal lexical processing, and this effect is modulated by reading skill. The findings are discussed in relation to the potential effect that enhanced parafoveal attention may have on everyday reading for deaf individuals.

## Introduction

Many studies employing low-level visual cognitive tasks suggest that, for deaf individuals, visual attention is reorganized to compensate for the lack or absence of auditory input provided by a complex environment [1], and, this reorganization has been thought to result in a redistribution of spatial visual attention toward the parafoveal and peripheral visual fields in deaf individuals [2, 3].

In support of this hypothesis, deaf individuals have been shown to respond more rapidly to flashed or moving stimuli in the periphery [4, 5], and they have been reported to have enhanced visual selective attention in the peripheral visual field [6]. There is also evidence from fMRI studies that the middle temporal-medial superior temporal area of deaf individuals shows a greater response to peripheral motion stimuli compared to individuals with typical levels of hearing [7, 8].

However, in some circumstances increased parafoveal or peripheral visual attention might hamper foveal visual field performance. For example, Proksch and Bavelier have shown that deaf individuals exhibit greater distractibility from parafoveal distractors in target detection tasks [9], and reduced discrimination performance was reported when a central discrimination task and a peripheral localization task had to be carried out simultaneously [10]. These studies, using low-level cognitive tasks, have shown that deaf individuals are more distracted by the presentation of task-irrelevant information in peripheral vision when the task requirements demand a central focus. An important question leading from these studies is, to what extent increased parafoveal processing in deaf individuals might impact an everyday task, such as reading.

It is well documented that the illiteracy rate of deaf individuals has been very high for decades. The average reading proficiency of young deaf adults graduating from high school has been reported to be far behind that of individuals with typical levels of hearing [11].

It is also well known that there is a strong link between visual attention and reading efficiency in individuals with typical levels of hearing [12]. For example, it has been shown that selective spatial attention is significantly associated with reading efficiency [13], and that visual-spatial attention can effectively predict reading achievement [14] in hearing individuals.

Foveal information (the central 2° of the visual field) is essential for word processing and for reading [15]. However, eye movement studies in reading have also indicated that linguistic information can be obtained from the parafoveal region (from the foveal region up to 5° of visual angle on either side of fixation). If readers’ allocate attentional resources to process parafoveal words, then reading efficiency can be increased, but, paradoxically, if too much attention is allocated to process parafoveal information, then this may actually hinder identification of the foveal word under inspection [16]. Therefore, readers have to carefully allocate their attention between the foveal region and parafoveal region, in order to obtain parafoveal information that is useful (facilitatory), but not harmful (inhibitory) for reading [17].

Given the relationship between foveal and parafoveal processing in reading, it has been speculated that the redistribution of spatial visual attention toward the parafoveal visual fields, observed for deaf individuals, may actually slow down foveal processing, and hence cause reading difficulties for deaf individuals [18]. In support of that view, some studies have shown that deaf readers, including Chinese deaf readers, can access parafoveal information more efficiently during sentence reading [19, 20]. However, although these studies show deaf individuals to have enhanced access to parafoveal information more efficiently during sentence reading, they cannot tell us how visual processing of parafoveal information may influence foveal lexical access during reading in deaf readers.

In this paper, we investigate whether the redistribution of spatial visual attention toward the parafoveal visual fields in deaf individuals impacts foveal lexical access for a higher-level cognitive task (lexical decision), with an ultimate aim of relating how the findings might affect the everyday task of reading.

The current study adopted the modified flanker paradigm [21] and utilized a lexical decision task to investigate the effects of task-irrelevant linguistic stimuli, presented in parafoveal locations, on central lexical processing in deaf individuals.

If the redistribution of spatial visual attention toward the parafoveal visual fields in deaf readers affects the lexical processing of stimuli under foveal inspection, it would be predicted that deaf participants should take longer to make a lexical decision when a target character is presented centrally along with a distractor character simultaneously presented in parafoveal vision. We, therefore, aimed to test whether interference effects were greater for deaf college student readers compared to participants with typical levels of hearing.

A further aim of this experiment was to explore whether any observed interference effects for the deaf readers were modulated by reading level ability. Although there is no empirical evidence to support that claim, it could be the case that high-skilled deaf readers are able to inhibit irrelevant distractors more effectively than low-skilled deaf readers. If this is the case we would predict a greater interference effect for low-skilled deaf readers compared to high-skilled deaf readers. One of the challenges in the field is to uncover factors that distinguish skilled deaf readers from unskilled deaf readers [22]. Control over the redistribution of spatial visual attention may be a factor that distinguishes between these two groups of deaf readers.

## Method

### Ethics statement

The procedures of the study have been approved by the Ethical Committee of Tianjin Normal University. All deaf college student participants and all college students with typical levels of hearing provided informed written consent prior to inclusion in the study. Parents provided informed written consent for middle school students with typical levels of hearing.

### Participants

Forty severely to profoundly deaf college students (*M*_age_ = 20.69 years, *SD* = 1.25) were recruited from the Technical College for the Deaf, Tianjin University of Technology, China. The deaf participants’ hearing losses were ≥ 75dB (*M* = 100.45 dB, *SD* = 12.61) in the better ear. Participants were either born deaf or became deaf before three years of age. None of the participants had received a cochlear implant. Thirty-five deaf participants reported sign language as their primary and preferred language of communication. Five deaf participants reported spoken language as their primary and preferred language of communication. Demographic information of participants was collected by a questionnaire survey. The information was provided by the participants themselves. The power analysis of the current study for an average effect size of *d* = 0.45 is 0.97, and that power value is greater than the minimum recommended level of 0.80 [23, 24].

A reading control group consisted of forty Chinese middle school students with typical levels of hearing (*M*_age_ = 13.98 years, *SD* = 0.79). We matched their reading comprehension level on the *Chinese Proficiency Test for Deaf College Students* [25, 26]. This test was developed for deaf college students and consists of three parts: sentence comprehension, short text comprehension, and ancient text comprehension. The test has a total of 50 multiple choice questions, with 2 points given for each question. The completion time is 30 minutes. There was no significant difference in reading comprehension scores between the deaf college students (*M* = 67.03, *SD* = 5.63) and the reading control group (*M* = 66.48, *SD* = 7.92), *t* (78) = 0.36, *p* = .72. There was no significant difference in reading fluency [20] scores between the deaf college students (*M* = 342.34 characters/min, *SD* = 111.98) and the reading control group (*M* = 330.78 characters/min, *SD* = 101.20), *t* (78) = 0.49, *p* = .63.

An age control group consisted of forty Chinese college students with typical levels of hearing (*M*_age_ = 20.49 years, *SD* = 1.24). There was no significant difference in chronological age between the deaf college students and the age control group, *t* (78) = – 0.71, *p* = .48. There was a significant difference in reading comprehension scores between the deaf college students (*M* = 67.03, *SD* = 5.63) and the age control group (*M* = 86.15, *SD* = 5.33), *t* (78) = 15.61, *p* < .001, *d* = 3.49 [25, 26]. There was a significant difference in reading fluency [20] scores between the deaf college students (*M* = 342.34 characters/min, *SD* = 111.98) and the age control group (*M* = 458.23 characters/min, *SD* = 89.58), *t* (78) = 5.11, *p* < .001, *d* = 1.14.

In addition, we matched participants from the three groups on nonverbal IQ using the Raven Test [27, 28]. There was no significant difference in standard score among the three groups, *F* (2,117) = 0.20, *p* = .82. All participants had normal or corrected-to-normal vision.

### Materials

There were 360 trials (see S1 Materials), of which 180 trials presented pseudo-Chinese characters and were used as filler materials [29, 30]; Eighty percent of trials were target-distractor pairs, and the other 20 percent of trials were without distractors.

For the target-distractor pairs, twenty undergraduate students with typical levels of hearing, who did not participate in the experiment, evaluated the relatedness of target characters and distractor characters on a 5 point scale (1 = *very dissimilar*, 5 = *very similar*). The targets and distractors shared no orthographic (*M* = 1.39, *SD* = 0.38), phonological (*M* = 1.19, *SD* = 0.26), or semantic (*M* = 1.37, *SD* = 0.38) similarities. The target characters mean frequency was 145.99 per million (*SD* = 430.01) and the distractor characters mean frequency was 120.72 per million (*SD* = 548.35), and the target characters and distractor characters mean number of strokes was 8.72 (*SD* = 3.23) and 8.74 (*SD* = 3.00), respectively [31].

Given that the parafoveal region is defined as up to 5° visual angle on either side of fixation, the parafoveal presentation locations for the study were operationalized as 2°, 3°, or 4° visual angle from the center of the display on either side. Half of the distractors were presented to the left parafoveal visual field and half to the right. The negative sign and positive sign represent the left and the right of the central fixation point, respectively.

### Design

The study used a 3 (Group: deaf college student group, reading control group, age control group) ×7 (Interference condition: without interference, –4°, –3°, –2°, + 2°, +3°, +4°) mixed experimental design. The group was a between-subjects factor and interference condition was a within-subjects factor.

### Procedure

Participants were seated 45cm from the 14-inch monitor of a laptop (with a 60-Hz refresh rate, 1024×768 resolution). The pixel value of each character was 34.67 × 34.67. At this distance, one character subtended about 1° visual angle [32]. A software package (Eprime2.0) was used to run the program and record reaction times (RTs).

**Fig 1:**
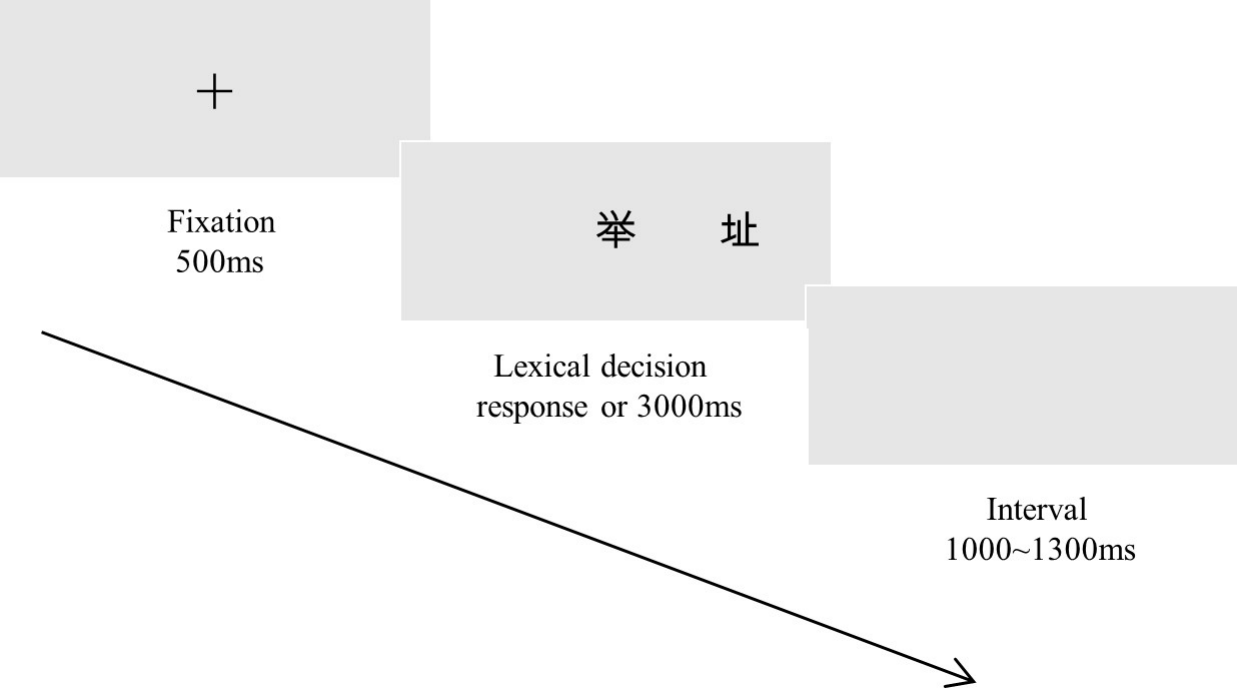
Example of distractor trial sequence. Fig 1 represents a trial of +3° interference condition. The target character (举, *lift*) was presented at the center of the screen and the distractor character (址, *address*) was presented at 3° visual angle of right side from the center of the display.

Each trial began with a cross at the center of the screen for 500ms. The target character was then presented at the center of the screen (with/without a distractor character being presented on either side of fixation at different locations, depending on the interference condition). Participants were asked to focus on the central fixation and were asked to make a lexical decision for the foveal target as quickly and accurately as possible when this was presented, regardless of whether a parafoveal distractor was simultaneously presented with the target. Participants used the right or left index finger to press the F or J on the keyboard, indicating character (word) or pseudo-character (non-word), respectively. Participants had a maximum of 3s to respond [32]. Each target presentation trial was followed by a random interval 1000~1300ms before the next trial sequence began; Fig 1 shows a schematic of the trial sequence. There was a break after every 72 trials and the experiment lasted approximately 30 minutes.

### Data analysis

The analysis of RTs excluded pseudo-Chinese character filler trials, incorrectly answered trials (4.35%), and trials with an RT which were beyond 2.5 standard deviations (2.82%) from each participant’s mean RT (see S1 Dataset). Analysis of error rates excluded pseudo-Chinese character filler trials and trials which were beyond 2.5 standard deviations (2.74%) from each participant’s mean RT [29, 30].

Data were analyzed using the lme4 package [33], within the R environment for statistical computing [34]. Generalized linear mixed models were used to analyze error rate data (binary variables) [35]. We report *b* values, standard errors (*SE*) and *t values (z values for* error rates *data)*, with |*t*| ≥ 1.96 (|*z*| ≥ 1.96) deemed significant at the 0.05 alpha level. In the model, participants and items were specified as crossed random effects [36]. Group and interference condition were specified as fixed effects. To assess differences between pairs of groups, we set up two successive difference contrasts: comparing the age control group to the deaf college student group, and the deaf college student group to the reading control group. Analysis of interference effects was based on planned comparisons for each of the different interference conditions and the without-interference (single target) condition, which was used as a reference condition. In this model, the interactions represent the interference effect for a given pair of groups. If the maximal random effects structure model failed to converge, the random structure was trimmed, and then we used a likelihood ratio test to verify the best fit of the converged models to the data [37].

## Results

### Error rates

When different interference conditions were compared to the without-interference condition separately, the random effects structure model failed to converge. Therefore, we compared different interference conditions as a whole to the without-interference condition and the model including participant and item random intercepts converged.

The deaf college student group average error rate was 3.63%; the age control group average error rate was 3.13%; the reading control group average error rate was 5.86%; See Table A in S1 Appendix.

As shown in Table 1, the deaf college student group did not differ in their error rate with the age control group (*b* = 0.20, *SE* = 0.19, *z* = 1.05); the deaf college student group and the reading control group was significantly different in error rate (*b* = 0.53, *SE* = 0.18, *z* = 2.96), and the interference effect main effect was not significant (*b* = 0.09, *SE* = 0.17, *z* = 0.51). The deaf college student group and the age control group did not significantly interact with the interference effect (*b* = – 0.11, *SE* = 0.23, *z* = – 0.48), and the deaf college student group and the reading control group did not significantly interact with the interference effect (*b* = 0.14, *SE* = 0.20, *z* = 0.69). Thus the only significant result for the error rate analysis, in relation to the deaf college student group, showed that the reading control group made more errors compared to the deaf college students, and this finding can most likely be explained by age differences between those two groups.

**Table 1:** Groups × interference conditions results for error rates (%).

	*b*	*SE*	*z*
Intercept	-3.61	0.11	**-34.11**
Deaf college student group vs. Age control group	0.20	0.19	1.05
Reading control group vs. Deaf college student group	0.53	0.18	**2.96**
Interference vs. Without interference	0.09	0.17	0.51
Deaf college student group vs. Age control group × Interference vs. Without interference	-0.11	0.23	-0.48
Reading control group vs. Deaf college student group × Interference vs. Without interference	0.14	0.20	0.69

*Note*. Deaf college student group: forty severely to profoundly deaf college students. Age control group: forty Chinese college students with typical levels of hearing. Reading control group: forty Chinese middle school students with typical levels of hearing. |*z*|≥ 1.96 deemed significant at the 0.05 alpha level. Significant *z*-values are shown in bold.

### Reaction times

A model that included participant random slope alongside participant and item random intercepts was deemed the best model for analysis, based on the likelihood-ratio test result [36].

As shown in Table 2, the group main effects did not differ: The deaf college student group did not differ in their RTs (see Table B in S1 Appendix) from the age control group (*b* = 26.03, *SE* = 28.78, *t* = 0.90); and the deaf college student group did not differ in their RTs from the reading control group (*b* = 1.48, *SE* = 28.78, *t* = 0.05). The interference effect main effects were significant in all interference conditions, all |*t*s| >1.96, showing that distractors presented at all eccentricities resulted in longer RTs compared to when no distractors were presented (the without distractor condition).

**Table 2:** Groups × interference conditions results for RTs (ms).

	*b*	*SE*	*t*
Intercept	654.93	12.07	**54.27**
Deaf college student group vs. Age control group	26.03	28.78	0.90
Reading control group vs. Deaf college student group	1.48	28.78	0.05
–2°vs.Without interference	-92.76	10.45	**-8.88**
–3°vs.Without interference	-79.64	11.01	**-7.24**
–4°vs.Without interference	-79.87	10.71	**-7.46**
+2°vs.Without interference	-79.28	10.13	**-7.83**
+3°vs.Without interference	-73.46	10.55	**-6.96**
+4°vs.Without interference	-74.70	10.51	**-7.11**
Deaf college student group vs. Age control group × –2°vs.Without interference	-59.62	19.38	**-3.08**
Reading control group vs. Deaf college student group × –2°vs.Without interference	58.12	19.46	**2.99**
Deaf college student group vs. Age control group × –3°vs.Without interference	-79.32	21.16	**-3.75**
Reading control group vs. Deaf college student group × –3°vs.Without interference	75.05	21.23	**3.53**
Deaf college student group vs. Age control group × –4°vs.Without interference	-88.90	20.23	**-4.39**
Reading control group vs. Deaf college student group× –4°vs.Without interference	86.14	20.30	**4.24**
Deaf college student group vs. Age control group × +2°vs.Without interference	-63.58	18.34	**-3.47**
Reading control group vs. Deaf college student group× +2°vs.Without interference	55.50	18.40	**3.02**
Deaf college student group vs. Age control group × +3°vs.Without interference	-83.47	19.73	**-4.23**
Reading control group vs. Deaf college student group × +3°vs.Without interference	88.39	19.80	**4.46**
Deaf college student group vs. Age control group × +4°vs.Without interference	-80.94	19.59	**-4.13**
Reading control group vs. Deaf college student group × +4°vs.Without interference	78.34	19.64	**3.99**

*Note*. Deaf college student group: forty severely to profoundly deaf college students. Age control group: forty Chinese college students with typical levels of hearing. Reading control group: forty Chinese middle school students with typical levels of hearing. Interference conditions: The negative sign and positive sign represent the left and the right of the central fixation point, respectively. |*t*| ≥ 1.96 deemed significant at the 0.05 alpha level. Significant *t-*values are shown in bold.

**Fig 2:**
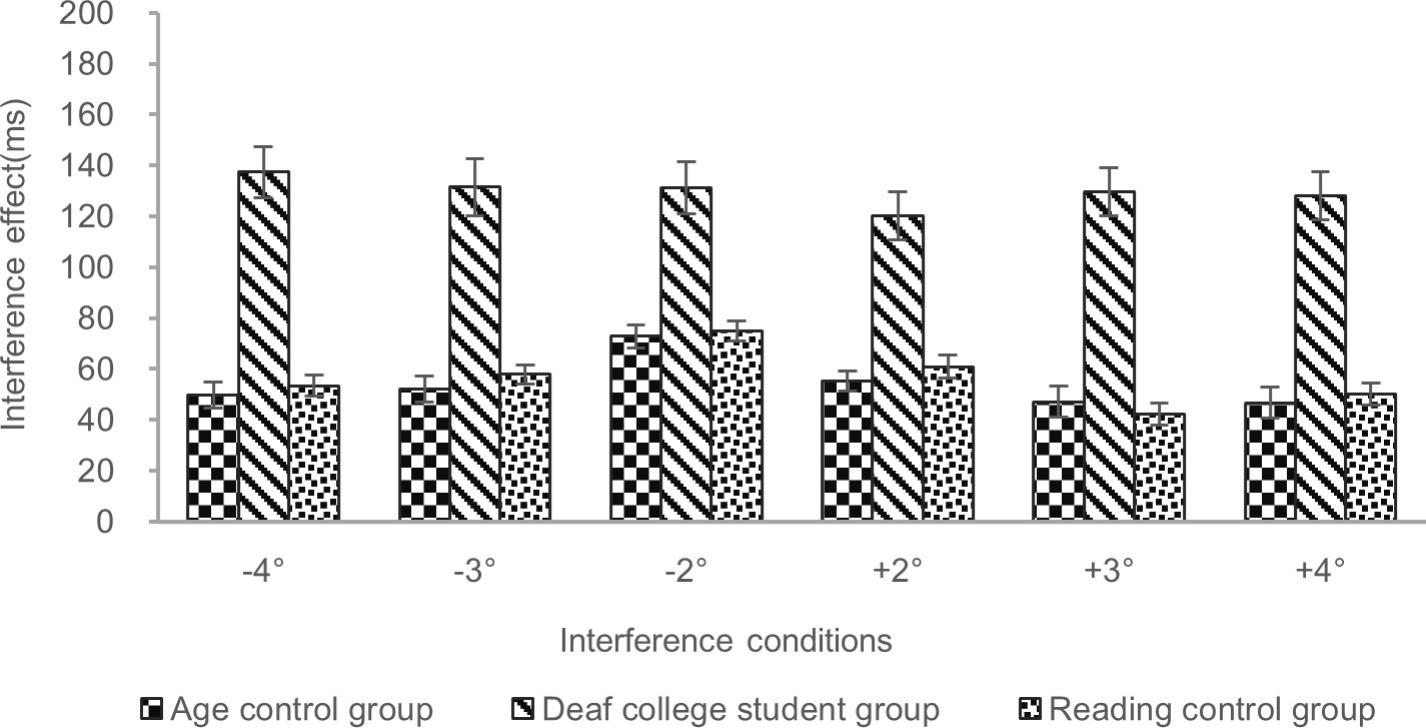
Comparison of the interference effect for three participant groups in different interference conditions. Deaf college student group: forty severely to profoundly deaf college students. Age control group: forty Chinese college students with typical levels of hearing. Reading control group: forty Chinese middle school students with typical levels of hearing. Interference conditions: The negative sign and positive sign represent the left and the right of the central fixation point, respectively. Interference effect (ms) = RTs with interference—RTs without interference (single target). Error bars represent the standard errors of the means.

As shown in Table 2, the deaf college student group and age control group significantly interacted with the interference effect in all interference conditions, all |*t*s| >1.96, indicating that the interference effect was larger for the deaf college student group than for age control group; see Fig 2. The deaf college student group and reading control group significantly interacted with the interference effect in all interference conditions, all |*t*s| >1.96, indicating that the interference effect was also larger for the deaf college student group than for the reading control group [20].

In summary, the results showed that the presence of a parafoveal lexical distractor resulted in poorer performance (longer RTs) for a lexical decision task in the deaf participants compared to the participants with typical levels of hearing.

A further aim of this study was to investigate whether reading skill modulated observed interference effects in deaf individuals. We tested this hypothesis by comparing interference effects between different reading levels of deaf individuals. If deaf individuals with low-skilled reading levels allocate more attentional resources to process parafoveal information than deaf individuals with high-skilled reading levels, we predicted that low-skilled deaf readers in the current study should have taken longer to make a lexical decision compared to high-skilled deaf readers in the current study.

## Analyses of interference effects for deaf readers with high-skilled reading levels versus deaf readers with low-skilled reading levels

We separated the deaf college students into individuals with high-skilled reading levels and individuals with low-skilled reading levels, using a median split technique [38] based on their reading comprehension [25, 26] test scores (Given that this test was developed for deaf college students, it can be argued to reflect and distinguish reading ability of deaf college students). The two groups comprised a low/medium skilled group (*n* = 19) with reading comprehension test scores < 68.00, and a high-skilled group (*n* = 21) with all scores ≥ 68.00. There was a significant difference in reading comprehension scores between the low-skilled deaf group (*M* = 62.05, *SD* = 3.37) and the high-skilled deaf group (*M* = 71.52, *SD* = 2.60), *t* (38) = 10.00, *p* < .001, *d* = 3.17. In the low-skilled deaf group, seventeen deaf participants reported sign language as their primary and preferred language of communication, and two deaf participants reported spoken language as their primary and preferred language of communication. In the high-skilled deaf group, eighteen deaf participants reported sign language as their primary and preferred language of communication, and three deaf participants reported spoken language as their primary and preferred language of communication.

In relation to the hearing loss of the deaf readers, there was no significant difference in the better ear between the low-skilled deaf group (*M* = 100.32 dB, *SD* = 12.13) and the high-skilled deaf group (*M* = 100.57 dB, *SD* = 13.32), *t* (38) = 0.06, *p* = .95. There was no significant difference in chronological age between the low-skilled deaf group (*M* = 20.71, *SD* = 1.42) and the high-skilled deaf group (*M* = 20.68, *SD* = 1.12), *t* (38) = – 0.09, *p* = .93. Finally, there was no significant difference in IQ standard score between the low-skilled deaf group (*M* = 101.32, *SD* = 14.99) and the high-skilled deaf group (*M* = 109.83, *SD* = 13.51), *t* (38) = 1.89, *p* = .07.

### Error rates

When different interference conditions were compared to the without-interference condition separately, the random effects structure model failed to converge. Therefore, we compared different interference conditions as a whole to the without-interference condition.

The low-skilled deaf group average error rate was 3.74%; the high-skilled deaf group average error rate was 3.53%; See Table C in S1 Appendix.

As shown in Table 3, the low-skilled deaf group did not differ in their error rate from the high-skilled deaf group (*b* = – 0.16, *SE* = 0.28, *z* = – 0.56). The interference effect main effect was not significant (*b* = – 0.03, *SE* = 0.27, *z* = – 0.13), and the low-skilled deaf group and the high-skilled deaf group did not significantly interact with the interference effect (*b* = 0.51, *SE* = 0.33, *z* = 1.54).

**Table 3:** Groups × interference conditions results for error rates (%).

	*b*	*SE*	*z*
Intercept	-3.94	0.19	**-21.00**
Low-skilled deaf group vs. High-skilled deaf group	-0.16	0.28	-0.56
Interference vs. Without interference	-0.03	0.27	-0.13
Low-skilled deaf group vs. High-skilled deaf group × Interference vs. Without interference	0.51	0.33	1.54

*Note*. Low-skilled deaf group: nineteen severely to profoundly deaf college students with reading comprehension test scores < 68.00. High-skilled deaf group: twenty-one severely to profoundly deaf college students with scores ≥ 68.00.|*z*|≥ 1.96 deemed significant at the 0.05 alpha level. Significant *z*-value is shown in bold.

### Reaction times

A model that included participant random slope alongside participant and item random intercepts was deemed the best model for analysis, based on the likelihood-ratio test result [36].

As shown in Table 4, the low-skilled deaf group had longer RTs (see Table D in S1 Appendix) than the high-skilled deaf group (*b* = 102.47, *SE* = 50.54, *t* = 2.03), and the interference effect main effects were significant in all interference conditions, all |*t*s| >1.96.

**Table 4:** Groups × interference conditions results for RTs (ms).

	*b*	*SE*	*t*
Intercept	666.07	25.48	**26.15**
Low-skilled deaf group vs. High-skilled deaf group	102.47	50.45	**2.03**
–2°vs.Without interference	–135.48	21.17	–**6.40**
–3°vs.Without interference	–133.41	23.57	–**5.66**
–4°vs.Without interference	–140.12	22.24	–**6.30**
+2°vs.Without interference	–121.20	20.27	–**5.98**
+3°vs.Without interference	–132.59	20.74	–**6.39**
+4°vs.Without interference	–130.25	20.60	–**6.32**
Low-skilled deaf group vs. High-skilled deaf group × –2°vs.Without interference	–82.65	38.47	–**2.15**
Low-skilled deaf group vs. High-skilled deaf group × –3°vs.Without interference	–76.38	43.70	–1.75
Low-skilled deaf group vs. High-skilled deaf group × –4°vs.Without interference	–61.14	40.81	–1.50
Low-skilled deaf group vs. High-skilled deaf group × +2°vs.Without interference	–85.66	36.49	–**2.35**
Low-skilled deaf group vs. High-skilled deaf group × +3°vs.Without interference	–63.67	37.51	–1.70
Low-skilled deaf group vs. High-skilled deaf group × +4°vs.Without interference	–70.38	37.20	–1.89

*Note*. Low-skilled deaf group: nineteen severely to profoundly deaf college students with reading comprehension test scores < 68.00.High-skilled deaf group: twenty-one severely to profoundly deaf college students with scores ≥ 68.00. Interference conditions: The negative sign and positive sign represent the left and the right of the central fixation point, respectively.|*t*| ≥ 1.96 deemed significant at the 0.05 alpha level. Significant *t-*values are shown in bold.

**Fig 3:**
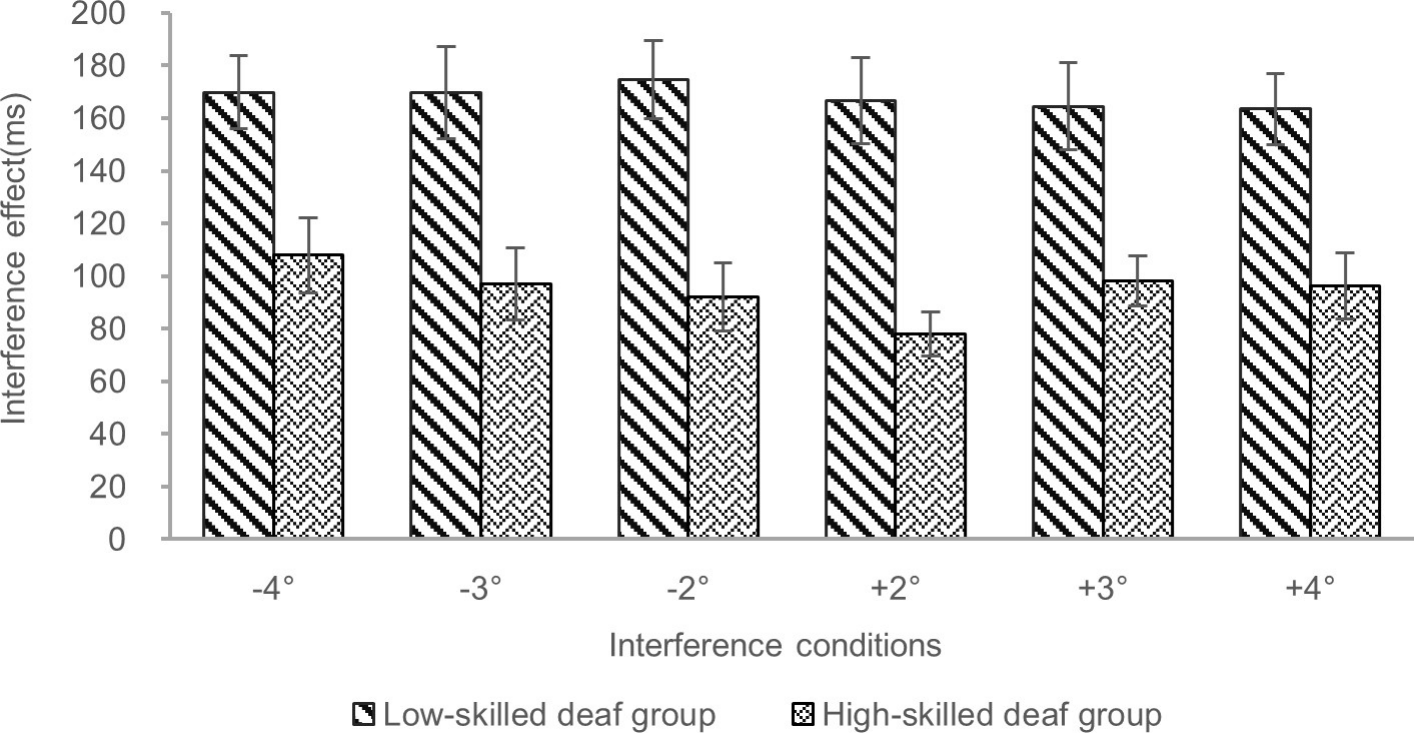
Comparison of the interference effect for two deaf reader groups in different interference conditions. Low-skilled deaf group: nineteen severely to profoundly deaf college students with reading comprehension test scores < 68.00. High-skilled deaf group: twenty-one severely to profoundly deaf college students with scores ≥ 68.00. Interference conditions: The negative sign and positive sign represent the left and the right of the central fixation point, respectively. Interference effect (ms) = RTs with interference—RTs without interference (single target). Error bars represent the standard errors of the means.

As shown in Table 4, the two groups of deaf readers significantly interacted with the interference effects for 2° conditions on either side of fixation; see Fig 3, indicating that the interference effect was larger for the low-skilled deaf group (for –2° vs. without interference, 171.63 ms; for +2° vs. without interference, 163.98 ms) than for the high-skilled deaf group (for –2°vs. without interference, 95.05 ms; for +2°vs. without interference, 78.07 ms). The two groups of deaf readers marginally significantly interacted with the interference effects for 3° interference conditions on either side of fixation and for the +4° interference condition, indicating that the interference effect was larger for the low-skilled deaf group (for –3° vs. without interference, 165.60 ms; for +3° vs. without interference, 161.15 ms; for +4° vs. without interference,163.10 ms) than for the high-skilled deaf group (for –3° vs. without interference, 95.59 ms; for +3° vs. without interference, 98.07 ms; for +4° vs. without interference, 98.36 ms) in the interference conditions. For the –4° interference condition, the low-skilled deaf group (for –4°vs. without interference,168.68 ms) show an obvious data trend of a larger interference effect than for the high-skilled deaf group (for –4° vs. without interference, 109.50 ms) but this numerical difference was not statistically significant [20].

In summary, separating the deaf students into high and low-skilled deaf readers resulted in differences in the magnitude of distractor effects on the time taken to make a lexical decision, with low-skilled readers taking longer than high-skilled readers. Since there were equivalent numbers of deaf students who rely on sign language as their preferred way to communicate, it is unlikely that this preference impacted on the modulating effects of reading skill on distractor interference.

One interesting question that arises from this finding relates to whether the increased interference effect for low-skilled readers is unique to deaf readers, or whether the same effect is observed for hearing readers? To address this question, we carried out the following analyses for the reading matched hearing participants.

## Analyses of interference effects for reading-matched hearing participants with high-skilled reading levels versus low-skilled reading levels

We separated the reading-matched hearing middle school participants into individuals with high-skilled reading levels and individuals with low-skilled reading levels, using a median split technique [38] based on their reading comprehension [25, 26] test scores. The two groups comprised a low/medium skilled group (*n* = 21) with reading comprehension test scores ≤ 64.00, and a high-skilled group (*n* = 19) with scores > 64.00. There was a significant difference in reading comprehension test scores between the low-skilled group (*M* = 60.38, *SD* = 3.44) and the high-skilled group (*M* = 73.21, *SD* = 5.61), *t* (38) = 8.81, *p*< .001, *d* = 2.79.

There was no significant difference of chronological age between the low-skilled group (*M* = 13.83, *SD* = 0.79) and the high-skilled group (*M* = 14.15, *SD* = 0.77), *t* (38) = 1.32, *p* = .20; and no significant difference in IQ standard score between the low-skilled group (*M* = 101.95, *SD* = 9.75) and the high-skilled group (*M* = 108.00, *SD* = 11.55), *t* (38) = 1.80, *p* = .08.

### Error rates

We compared different interference conditions as a whole to the without-interference condition. The average error rate of the low-skilled group was 6.67%; the average error rate of the high-skilled group was 4.94%; See Table E in S1 Appendix.

As shown in Table 5, the low-skilled group did not differ in their error rate from the high-skilled group (*b* = 0.39, *SE* = 0.22, *z* = 1.81). There was no significant main effect of interference (*b* = 0.14, *SE* = 0.19, *z* = 0.71), and the low-skilled group and the high-skilled group did not significantly interact with the interference effect (*b* = 0.00, *SE* = 0.26, *z* = -0.02).

**Table 5:** Groups × interference conditions results for error rates (%).

	*b*	*SE*	*z*
Intercept	-3.17	0.13	**-23.65**
Low-skilled group vs. High-skilled group	0.39	0.22	1.81
Interference vs. Without interference	0.14	0.19	0.71
Low-skilled group vs. High-skilled group × Interference vs. Without interference	0.00	0.26	-0.02

*Note*. Low-skilled group: twenty-one hearing middle school participants with reading comprehension test scores ≤ 64.00. High-skilled group: nineteen hearing middle school participants with scores > 64.00.|*z*|≥ 1.96 deemed significant at the 0.05 alpha level. Significant *z*-value is shown in bold.

### Reaction times

The maximal random effects structure, including participant and item random intercepts, converged. As shown in Table 6, the low-skilled group did not differ in their RTs (see Table F in S1 Appendix) from the high-skilled group (*b* = 33.30, *SE* = 28.20, *t* = 1.18), and the interference effect main effects were significant in all interference conditions, all |*t*s| >1.96.

**Table 6:** Groups × interference conditions results for RTs (ms).

	*b*	*SE*	*t*
Intercept	663.30	14.34	**46.26**
Low-skilled group vs. High-skilled group	33.30	28.20	1.18
–2°vs.Without interference	-74.93	9.22	**-8.13**
–3°vs.Without interference	-57.62	9.21	**-6.25**
–4°vs.Without interference	-53.22	9.21	**-5.78**
+2°vs.Without interference	-63.91	9.21	**-6.94**
+3°vs.Without interference	-42.98	9.20	**-4.67**
+4°vs.Without interference	-48.87	9.18	**-5.33**
Low-skilled group vs. High-skilled group × –2°vs.Without interference	-7.65	13.14	-0.58
Low-skilled group vs. High-skilled group × –3°vs.Without interference	-4.50	13.15	-0.34
Low-skilled group vs. High-skilled group × –4°vs.Without interference	5.29	13.11	0.40
Low-skilled group vs. High-skilled group × +2°vs.Without interference	-21.71	13.14	-1.65
Low-skilled group vs. High-skilled group × +3°vs.Without interference	-22.20	13.09	-1.70
Low-skilled group vs. High-skilled group × +4°vs.Without interference	-28.10	13.02	**-2.16**

*Note*. Low-skilled group: twenty-one hearing middle school participants with reading comprehension test scores ≤ 64.00. High-skilled group: nineteen hearing middle school participants with scores > 64.00. Interference conditions: The negative sign and positive sign represent the left and the right of the central fixation point, respectively.|*t*| ≥ 1.96 deemed significant at the 0.05 alpha level. Significant *t-*values are shown in bold.

**Fig 4:**
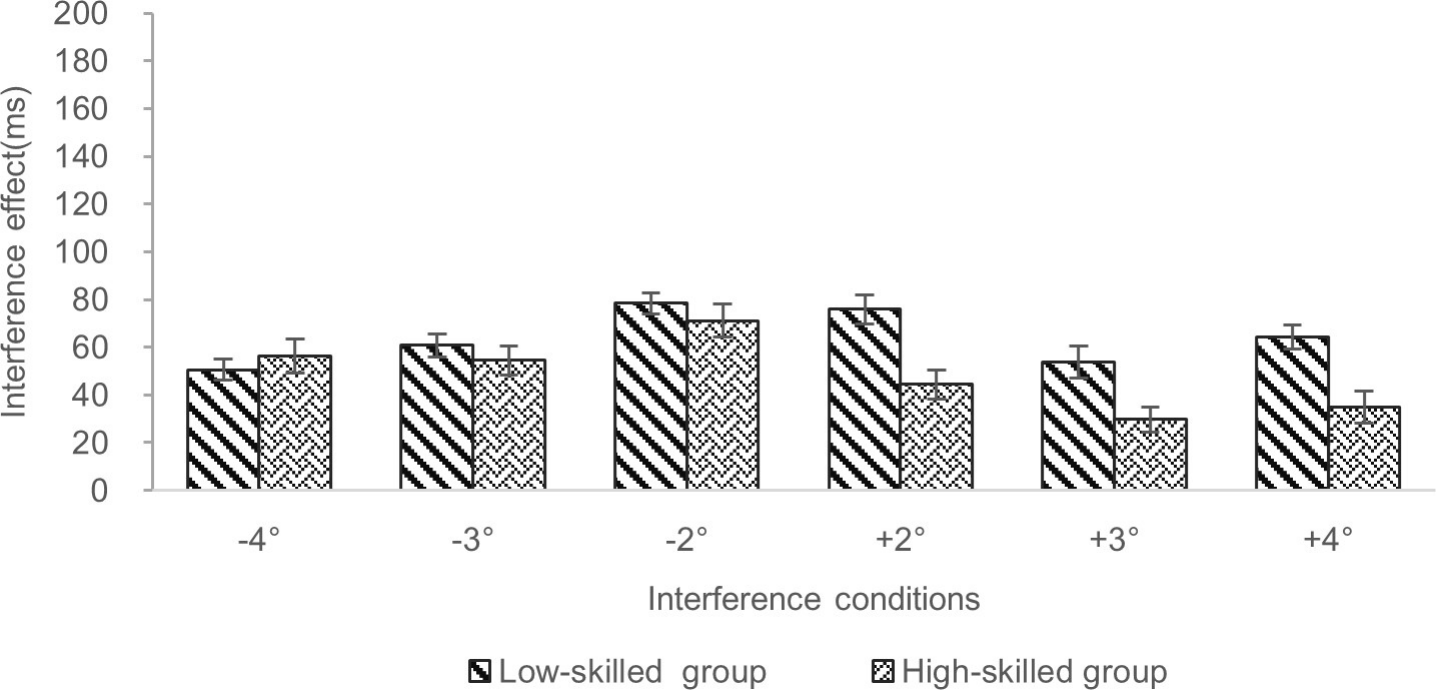
Comparison of the interference effect for two hearing middle school reader groups in different interference conditions. Low-skilled group: twenty-one hearing middle school participants with reading comprehension test scores ≤ 64.00. High-skilled group: nineteen hearing middle school participants with the scores > 64.00. Interference conditions: The negative sign and positive sign represent the left and the right of the central fixation point, respectively. Interference effect (ms) = RTs with interference—RTs without interference (single target). Error bars represent the standard errors of the means.

As shown in Table 6, the low-skilled group and the high-skilled group did not significantly interact with the interference effect in five condtions (|*t*s| < 1.96). The two groups of middle school participants significantly interacted with the interference effects for +4° condition; see Fig 4, indicating that the interference effect was larger for the low-skilled group (for +4° vs. without interference, 64.20 ms) than for the high-skilled group (for +4° vs. without interference, 34.74 ms). For the +2° and +3° interference conditions, the low-skilled group (for +2° vs. without interference, 76.30 ms; +3° vs. without interference, 54.66 ms) show a data trend of a larger interference effect than for the high-skilled group (for +2° vs. without interference, 50.08 ms; +3° vs. without interference, 32.18 ms), but this numerical difference was not statistically significant [20].

The pattern for the comparison between interference effects observed in the high-skilled and low-skilled deaf students appears to be different to that observed for the same comparison in the hearing students. In the deaf students interference effects are greater for the low-skilled readers when distractors are presented at different parafoveal eccentricities both to the right and to the left of fixation. However, for the hearing students, greater interference effects for the low-skilled readers in that group are obsreved exclusiveley for distractors presented to the right of fixation.

## Discussion

The aim of this study was to investigate whether the well documented redistribution of spatial visual attention toward the parafoveal visual fields in deaf individuals would influence the time taken to process lexical stimuli under foveal inspection. Firstly, we predicted that deaf participants should take longer to make a lexical decision when a target character was presented centrally along with a distractor character simultaneously presented in parafoveal vision. Secondly, we proposed that low-skilled deaf readers would show a greater interference effect compared to high-skilled deaf readers.

The results support our experimental hypotheses. We found a larger parafoveal interference effect for deaf college students (overall) than for participants with typical levels of hearing. We interpreted this finding to result from deaf individuals allocating more attentional resources to process parafoveal information. Hence, the redistribution of spatial visual attention in the deaf readers affects lexical processing of stimuli under foveal inspection.

In reading, the goal is to process the individual words sequentially and to integrate the meaning of these words in order to build up a full representation and comprehensive understanding of the text [39]. If readers’ allocate attentional resources to process parafoveal words, then reading efficiency can be increased, but, if too much attention is allocated to process parafoveal information, then this may actually hinder identification of the foveal word under inspection [16]. Therefore, readers have to carefully allocate their attention between the foveal region and parafoveal region, in order to obtain parafoveal information that is useful (facilitatory), but not harmful (inhibitory) for the reading process [17].

Some studies have found that deaf individuals can access parafoveal information more efficiently during sentence reading [19, 20], however, little is yet known about how visual processing of parafoveal information may influence foveal lexical access during reading for deaf readers. The findings from the current study support the hypothesis of the redistribution of spatial visual attention toward the parafoveal visual fields in deaf individuals. An obvious question raised by the findings would be to investigate whether the observed slow-down of foveal processing for lexical decision tasks could impact in everyday reading difficulties in deaf individuals, and whether any difficulties would be modulated by reading level in the deaf individuals.

However, it should also be noted that in normal reading parafoveal information is often related to foveal information, and hence has the potential to speed foveal processing. In that case it is possible that deaf readers might show a larger priming effect than hearing readers for parafoveal facilitatory information. Since the current study only tested an interfering stimulus (an unrelated character), the question as to whether greater parafoveal attention could enhance reading processes in the deaf readers remains to be tested.

Furthermore, the interference effects observed for the deaf students were modulated by reading level. Low-skilled deaf readers showed a greater interference effect compared to high-skilled deaf readers. This finding suggests that there may be differences in the way that low-skilled reading deaf individuals and high-skilled reading deaf individuals allocate attentional resources to parafoveal information.

In order to test whether that finding is unique to deaf readers, we also separated the reading-matched hearing middle school participants into individuals with high-skilled reading levels and individuals with low-skilled reading levels. The low-skilled middle school participants showed a greater interference effect (although not all effects were significant) compared to high-skilled middle school readers in interference conditions where distractors were presented to the right of fixation. This finding is observed exclusively for distractors presented to the right of fixation in low-skilled hearing individuals, whereas the effect is observed for distractors presented to the left and to the right of fixation in the low-skilled deaf readers. We interpret this finding to reflect a general effect of reading skill on modulating attention to the parafovea, but also that this modulation will be different for hearing and deaf individuals. A study of lexical decision flanker tasks with hearing readers has revealed a rightward bias that is argued to be related to reading direction [30], and in sentence reading tasks, the size of the perceptual span is asymmetric [15, 17] to the right for hearing individuals. Therefore, it is likely that an effect of reading skill would be detected for distractors presented to the right of fixation for hearing readers, but for deaf individuals an effect of reading skill would be detected for distractors presented to the right and the left of fixation. Compared with hearing readers, deaf individuals allocate more attentional resources to process parafoveal information presented to the left and to the right of fixation. This is in line with the findings from the current lexical decision study which clearly demonstrates an effect of reading skill on the ability to ignore parafoveal distractors, both to the left and to the right of fixation, for deaf readers.

A related finding from a case study of a hearing reader who was diagnosed with developmental dyslexia showed that the main reason for his reading problem was a selective attentional deficit, whereby letters from words available in parafoveal vision interfered with his processing of the currently fixated word [12]. This finding is similar to the results from the current study for deaf readers.

An important question raised by the findings relates to how the ability to control what is attended to and processed in the parafovea might affect reading skill development in deaf individuals.

## Conclusion

In sum, the current study confirms that deaf readers show a larger interference effect from irrelevant parafoveal distractors, during a lexical decision task. This is likely to result from the greater distribution of spatial visual attention toward the parafoveal visual field. Furthermore, low-skilled deaf readers show a larger interference effect which suggests that the allocation of attentional resources toward the parafovea, may reflect less volitional control in low-skilled deaf readers. The findings lead us to speculate that in deaf individuals, the ability to control what is processed or inhibited in the parafovea has the potential to impact in everyday reading in the deaf population.

## Supporting information

S1 Appendix (DOCX)

S1 Dataset Compiled raw data used for analysis the current study. (XLSX)

S1 Materials Materials used in the experiment. (ZIP)
