# Supplementary material for: Reading skill modulates the effect of parafoveal distractors on foveal lexical decision in deaf students
Source: PLoS One. 2019 Sep 12;14(9):e0221891. doi: 10.1371/journal.pone.0221891 (PMC6742358; doi:10.1371/journal.pone.0221891)
Supplement: S1 Appendix — (DOCX) [file pone.0221891.s001.docx]

**Table A. Error rate (%) in different conditions for the three participant groups.**

| Group | –4° | –3° | –2° | Without interference | +2° | +3° | +4° |
| --- | --- | --- | --- | --- | --- | --- | --- |
| Age control group | 3.50 | 2.45 | 2.48 | 2.97 | 4.13 | 3.42 | 3.02 |
| Deaf college student group | 3.28 | 4.00 | 3.96 | 3.75 | 3.11 | 3.30 | 3.94 |
| Reading control group | 6.75 | 6.67 | 5.75 | 5.46 | 5.76 | 6.02 | 4.80 |

*Note.* Deaf college student group: forty severely to profoundly deaf college students. Age control group: forty Chinese college students with typical levels of hearing. Reading control group: forty Chinese middle school students with typical levels of hearing. Interference conditions: The negative sign and positive sign represent the left and the right of the central fixation point, respectively.

**Table B. RTs (ms) in different** **conditions for the three participant groups.**

| Group | –4° | –3° | –2° | Without interference | +2° | +3° | +4° |
| --- | --- | --- | --- | --- | --- | --- | --- |
| Age control group | 638.15 | 639.74 | 659.70 | 589.63 | 644.61 | 636.49 | 637.82 |
| Deaf college student group | 687.26 | 678.87 | 681.09 | 550.00 | 668.97 | 677.58 | 678.26 |
| Reading control group | 668.24 | 674.75 | 691.42 | 616.28 | 680.08 | 660.34 | 666.59 |

*Note.* Deaf college student group: forty severely to profoundly deaf college students. Age control group: forty Chinese college students with typical levels of hearing. Reading control group: forty Chinese middle school students with typical levels of hearing. Interference conditions: The negative sign and positive sign represent the left and the right of the central fixation point, respectively.

**Table C. Error rates (%) in different conditions for the two deaf reader groups.**

| Group | –4° | –3° | –2° | Without interference | +2° | +3° | +4° |
| --- | --- | --- | --- | --- | --- | --- | --- |
| Low-skilled deaf group | 3.91 | 3.42 | 4.50 | 3.13 | 2.49 | 4.04 | 4.97 |
| High-skilled deaf group | 2.71 | 4.53 | 3.46 | 4.31 | 3.67 | 2.64 | 3.02 |

*Note.* Low-skilled deaf group: nineteen severely to profoundly deaf college students with reading comprehension test scores < 68.00. High-skilled deaf group : twenty-one severely to profoundly deaf college students with scores ≥ 68.00.Interference conditions: The negative sign and positive sign represent the left and the right of the central fixation point, respectively.

**Table D. RTs (ms) in different conditions for the two deaf reader groups.**

| Group | –4° | –3° | –2° | Without interference | +2° | +3° | +4° |
| --- | --- | --- | --- | --- | --- | --- | --- |
| Low-skilled deaf group | 740.94 | 737.87 | 743.90 | 572.27 | 736.25 | 733.42 | 735.37 |
| High-skilled deaf group | 639.12 | 625.21 | 624.68 | 529.62 | 607.69 | 627.69 | 627.98 |

*Note.* Low-skilled deaf group: nineteen severely to profoundly deaf college students with reading comprehension test scores < 68.00.High-skilled deaf group : twenty-one severely to profoundly deaf college students with scores ≥ 68.00. Interference conditions: The negative sign and positive sign represent the left and the right of the central fixation point, respectively.

**Table E. Error rates (%) in different conditions for the two middle school participant groups.**

| Group | –4° | –3° | –2° | Without interference | +2° | +3° | +4° |
| --- | --- | --- | --- | --- | --- | --- | --- |
| Low-skilled group | 6.98 | 7.20 | 7.08 | 6.22 | 7.28 | 6.80 | 5.34 |
| High-skilled group | 6.50 | 6.07 | 4.24 | 4.59 | 4.04 | 5.14 | 4.19 |

*Note.* Low-skilled group: twenty-one hearing middle school participants with reading comprehension test scores ≤ 64.00.High-skilled group : nineteen hearing middle school participants with scores > 64.00.Interference conditions: The negative sign and positive sign represent the left and the right of the central fixation point, respectively.

**Table F. RTs (ms) in different conditions for the two middle school participant groups.**

| Group | –4° | –3° | –2° | Without interference | +2° | +3° | +4° |
| --- | --- | --- | --- | --- | --- | --- | --- |
| Low-skilled group | 673.80 | 683.72 | 701.85 | 623.79 | 700.09 | 678.44 | 687.98 |
| High-skilled group | 661.96 | 664.58 | 679.87 | 607.92 | 658.00 | 640.10 | 642.66 |

*Note.* Low-skilled group: twenty-one hearing middle school participants with reading comprehension test scores ≤ 64.00.High-skilled group : nineteen hearing middle school participants with scores > 64.00.Interference conditions: The negative sign and positive sign represent the left and the right of the central fixation point, respectively.
