# Supplementary figures and images for: Reading skill modulates the effect of parafoveal distractors on foveal lexical decision in deaf students (part 2 of 2)
Source: PLoS One. 2019 Sep 12;14(9):e0221891. doi: 10.1371/journal.pone.0221891 (PMC6742358; doi:10.1371/journal.pone.0221891)

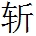

Supplement: S1 Materials — (ZIP) [file pone.0221891.s003.zip › S1_Materials/Dpseudoú¿144ú⌐/Dpseudo (6).jpg]

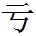

Supplement: S1 Materials — (ZIP) [file pone.0221891.s003.zip › S1_Materials/Dpseudoú¿144ú⌐/Dpseudo (60).jpg]

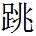

Supplement: S1 Materials — (ZIP) [file pone.0221891.s003.zip › S1_Materials/Dpseudoú¿144ú⌐/Dpseudo (61).jpg]

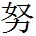

Supplement: S1 Materials — (ZIP) [file pone.0221891.s003.zip › S1_Materials/Dpseudoú¿144ú⌐/Dpseudo (62).jpg]

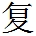

Supplement: S1 Materials — (ZIP) [file pone.0221891.s003.zip › S1_Materials/Dpseudoú¿144ú⌐/Dpseudo (63).jpg]

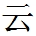

Supplement: S1 Materials — (ZIP) [file pone.0221891.s003.zip › S1_Materials/Dpseudoú¿144ú⌐/Dpseudo (64).jpg]

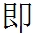

Supplement: S1 Materials — (ZIP) [file pone.0221891.s003.zip › S1_Materials/Dpseudoú¿144ú⌐/Dpseudo (65).jpg]

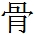

Supplement: S1 Materials — (ZIP) [file pone.0221891.s003.zip › S1_Materials/Dpseudoú¿144ú⌐/Dpseudo (66).jpg]

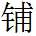

Supplement: S1 Materials — (ZIP) [file pone.0221891.s003.zip › S1_Materials/Dpseudoú¿144ú⌐/Dpseudo (67).jpg]

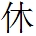

Supplement: S1 Materials — (ZIP) [file pone.0221891.s003.zip › S1_Materials/Dpseudoú¿144ú⌐/Dpseudo (68).jpg]

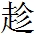

Supplement: S1 Materials — (ZIP) [file pone.0221891.s003.zip › S1_Materials/Dpseudoú¿144ú⌐/Dpseudo (69).jpg]

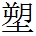

Supplement: S1 Materials — (ZIP) [file pone.0221891.s003.zip › S1_Materials/Dpseudoú¿144ú⌐/Dpseudo (7).jpg]

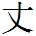

Supplement: S1 Materials — (ZIP) [file pone.0221891.s003.zip › S1_Materials/Dpseudoú¿144ú⌐/Dpseudo (70).jpg]

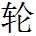

Supplement: S1 Materials — (ZIP) [file pone.0221891.s003.zip › S1_Materials/Dpseudoú¿144ú⌐/Dpseudo (71).jpg]

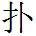

Supplement: S1 Materials — (ZIP) [file pone.0221891.s003.zip › S1_Materials/Dpseudoú¿144ú⌐/Dpseudo (72).jpg]

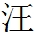

Supplement: S1 Materials — (ZIP) [file pone.0221891.s003.zip › S1_Materials/Dpseudoú¿144ú⌐/Dpseudo (73).jpg]

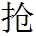

Supplement: S1 Materials — (ZIP) [file pone.0221891.s003.zip › S1_Materials/Dpseudoú¿144ú⌐/Dpseudo (74).jpg]

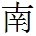

Supplement: S1 Materials — (ZIP) [file pone.0221891.s003.zip › S1_Materials/Dpseudoú¿144ú⌐/Dpseudo (75).jpg]

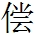

Supplement: S1 Materials — (ZIP) [file pone.0221891.s003.zip › S1_Materials/Dpseudoú¿144ú⌐/Dpseudo (76).jpg]

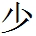

Supplement: S1 Materials — (ZIP) [file pone.0221891.s003.zip › S1_Materials/Dpseudoú¿144ú⌐/Dpseudo (77).jpg]

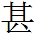

Supplement: S1 Materials — (ZIP) [file pone.0221891.s003.zip › S1_Materials/Dpseudoú¿144ú⌐/Dpseudo (78).jpg]

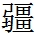

Supplement: S1 Materials — (ZIP) [file pone.0221891.s003.zip › S1_Materials/Dpseudoú¿144ú⌐/Dpseudo (79).jpg]

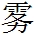

Supplement: S1 Materials — (ZIP) [file pone.0221891.s003.zip › S1_Materials/Dpseudoú¿144ú⌐/Dpseudo (8).jpg]

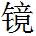

Supplement: S1 Materials — (ZIP) [file pone.0221891.s003.zip › S1_Materials/Dpseudoú¿144ú⌐/Dpseudo (80).jpg]

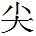

Supplement: S1 Materials — (ZIP) [file pone.0221891.s003.zip › S1_Materials/Dpseudoú¿144ú⌐/Dpseudo (81).jpg]

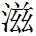

Supplement: S1 Materials — (ZIP) [file pone.0221891.s003.zip › S1_Materials/Dpseudoú¿144ú⌐/Dpseudo (82).jpg]

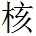

Supplement: S1 Materials — (ZIP) [file pone.0221891.s003.zip › S1_Materials/Dpseudoú¿144ú⌐/Dpseudo (83).jpg]

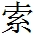

Supplement: S1 Materials — (ZIP) [file pone.0221891.s003.zip › S1_Materials/Dpseudoú¿144ú⌐/Dpseudo (84).jpg]

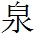

Supplement: S1 Materials — (ZIP) [file pone.0221891.s003.zip › S1_Materials/Dpseudoú¿144ú⌐/Dpseudo (85).jpg]

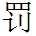

Supplement: S1 Materials — (ZIP) [file pone.0221891.s003.zip › S1_Materials/Dpseudoú¿144ú⌐/Dpseudo (86).jpg]

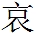

Supplement: S1 Materials — (ZIP) [file pone.0221891.s003.zip › S1_Materials/Dpseudoú¿144ú⌐/Dpseudo (87).jpg]

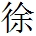

Supplement: S1 Materials — (ZIP) [file pone.0221891.s003.zip › S1_Materials/Dpseudoú¿144ú⌐/Dpseudo (88).jpg]

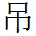

Supplement: S1 Materials — (ZIP) [file pone.0221891.s003.zip › S1_Materials/Dpseudoú¿144ú⌐/Dpseudo (89).jpg]

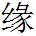

Supplement: S1 Materials — (ZIP) [file pone.0221891.s003.zip › S1_Materials/Dpseudoú¿144ú⌐/Dpseudo (9).jpg]

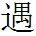

Supplement: S1 Materials — (ZIP) [file pone.0221891.s003.zip › S1_Materials/Dpseudoú¿144ú⌐/Dpseudo (90).jpg]

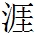

Supplement: S1 Materials — (ZIP) [file pone.0221891.s003.zip › S1_Materials/Dpseudoú¿144ú⌐/Dpseudo (91).jpg]

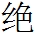

Supplement: S1 Materials — (ZIP) [file pone.0221891.s003.zip › S1_Materials/Dpseudoú¿144ú⌐/Dpseudo (92).jpg]

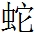

Supplement: S1 Materials — (ZIP) [file pone.0221891.s003.zip › S1_Materials/Dpseudoú¿144ú⌐/Dpseudo (93).jpg]

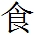

Supplement: S1 Materials — (ZIP) [file pone.0221891.s003.zip › S1_Materials/Dpseudoú¿144ú⌐/Dpseudo (94).jpg]

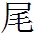

Supplement: S1 Materials — (ZIP) [file pone.0221891.s003.zip › S1_Materials/Dpseudoú¿144ú⌐/Dpseudo (95).jpg]

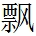

Supplement: S1 Materials — (ZIP) [file pone.0221891.s003.zip › S1_Materials/Dpseudoú¿144ú⌐/Dpseudo (96).jpg]

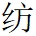

Supplement: S1 Materials — (ZIP) [file pone.0221891.s003.zip › S1_Materials/Dpseudoú¿144ú⌐/Dpseudo (97).jpg]

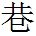

Supplement: S1 Materials — (ZIP) [file pone.0221891.s003.zip › S1_Materials/Dpseudoú¿144ú⌐/Dpseudo (98).jpg]

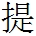

Supplement: S1 Materials — (ZIP) [file pone.0221891.s003.zip › S1_Materials/Dpseudoú¿144ú⌐/Dpseudo (99).jpg]

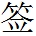

Supplement: S1 Materials — (ZIP) [file pone.0221891.s003.zip › S1_Materials/Drealú¿144ú⌐/Dreal (1).jpg]

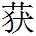

Supplement: S1 Materials — (ZIP) [file pone.0221891.s003.zip › S1_Materials/Drealú¿144ú⌐/Dreal (10).jpg]

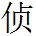

Supplement: S1 Materials — (ZIP) [file pone.0221891.s003.zip › S1_Materials/Drealú¿144ú⌐/Dreal (100).jpg]

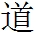

Supplement: S1 Materials — (ZIP) [file pone.0221891.s003.zip › S1_Materials/Drealú¿144ú⌐/Dreal (101).jpg]

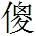

Supplement: S1 Materials — (ZIP) [file pone.0221891.s003.zip › S1_Materials/Drealú¿144ú⌐/Dreal (102).jpg]

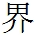

Supplement: S1 Materials — (ZIP) [file pone.0221891.s003.zip › S1_Materials/Drealú¿144ú⌐/Dreal (103).jpg]

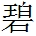

Supplement: S1 Materials — (ZIP) [file pone.0221891.s003.zip › S1_Materials/Drealú¿144ú⌐/Dreal (104).jpg]

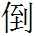

Supplement: S1 Materials — (ZIP) [file pone.0221891.s003.zip › S1_Materials/Drealú¿144ú⌐/Dreal (105).jpg]

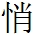

Supplement: S1 Materials — (ZIP) [file pone.0221891.s003.zip › S1_Materials/Drealú¿144ú⌐/Dreal (106).jpg]

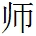

Supplement: S1 Materials — (ZIP) [file pone.0221891.s003.zip › S1_Materials/Drealú¿144ú⌐/Dreal (107).jpg]

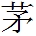

Supplement: S1 Materials — (ZIP) [file pone.0221891.s003.zip › S1_Materials/Drealú¿144ú⌐/Dreal (108).jpg]

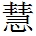

Supplement: S1 Materials — (ZIP) [file pone.0221891.s003.zip › S1_Materials/Drealú¿144ú⌐/Dreal (109).jpg]

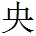

Supplement: S1 Materials — (ZIP) [file pone.0221891.s003.zip › S1_Materials/Drealú¿144ú⌐/Dreal (11).jpg]

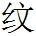

Supplement: S1 Materials — (ZIP) [file pone.0221891.s003.zip › S1_Materials/Drealú¿144ú⌐/Dreal (110).jpg]

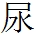

Supplement: S1 Materials — (ZIP) [file pone.0221891.s003.zip › S1_Materials/Drealú¿144ú⌐/Dreal (111).jpg]

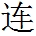

Supplement: S1 Materials — (ZIP) [file pone.0221891.s003.zip › S1_Materials/Drealú¿144ú⌐/Dreal (112).jpg]

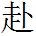

Supplement: S1 Materials — (ZIP) [file pone.0221891.s003.zip › S1_Materials/Drealú¿144ú⌐/Dreal (113).jpg]

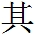

Supplement: S1 Materials — (ZIP) [file pone.0221891.s003.zip › S1_Materials/Drealú¿144ú⌐/Dreal (114).jpg]

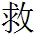

Supplement: S1 Materials — (ZIP) [file pone.0221891.s003.zip › S1_Materials/Drealú¿144ú⌐/Dreal (115).jpg]

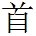

Supplement: S1 Materials — (ZIP) [file pone.0221891.s003.zip › S1_Materials/Drealú¿144ú⌐/Dreal (116).jpg]

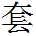

Supplement: S1 Materials — (ZIP) [file pone.0221891.s003.zip › S1_Materials/Drealú¿144ú⌐/Dreal (117).jpg]

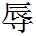

Supplement: S1 Materials — (ZIP) [file pone.0221891.s003.zip › S1_Materials/Drealú¿144ú⌐/Dreal (118).jpg]

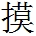

Supplement: S1 Materials — (ZIP) [file pone.0221891.s003.zip › S1_Materials/Drealú¿144ú⌐/Dreal (119).jpg]

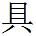

Supplement: S1 Materials — (ZIP) [file pone.0221891.s003.zip › S1_Materials/Drealú¿144ú⌐/Dreal (12).jpg]

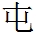

Supplement: S1 Materials — (ZIP) [file pone.0221891.s003.zip › S1_Materials/Drealú¿144ú⌐/Dreal (120).jpg]

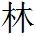

Supplement: S1 Materials — (ZIP) [file pone.0221891.s003.zip › S1_Materials/Drealú¿144ú⌐/Dreal (121).jpg]

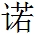

Supplement: S1 Materials — (ZIP) [file pone.0221891.s003.zip › S1_Materials/Drealú¿144ú⌐/Dreal (122).jpg]

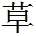

Supplement: S1 Materials — (ZIP) [file pone.0221891.s003.zip › S1_Materials/Drealú¿144ú⌐/Dreal (123).jpg]

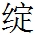

Supplement: S1 Materials — (ZIP) [file pone.0221891.s003.zip › S1_Materials/Drealú¿144ú⌐/Dreal (124).jpg]

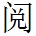

Supplement: S1 Materials — (ZIP) [file pone.0221891.s003.zip › S1_Materials/Drealú¿144ú⌐/Dreal (125).jpg]

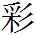

Supplement: S1 Materials — (ZIP) [file pone.0221891.s003.zip › S1_Materials/Drealú¿144ú⌐/Dreal (126).jpg]

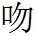

Supplement: S1 Materials — (ZIP) [file pone.0221891.s003.zip › S1_Materials/Drealú¿144ú⌐/Dreal (127).jpg]

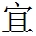

Supplement: S1 Materials — (ZIP) [file pone.0221891.s003.zip › S1_Materials/Drealú¿144ú⌐/Dreal (128).jpg]

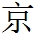

Supplement: S1 Materials — (ZIP) [file pone.0221891.s003.zip › S1_Materials/Drealú¿144ú⌐/Dreal (129).jpg]

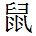

Supplement: S1 Materials — (ZIP) [file pone.0221891.s003.zip › S1_Materials/Drealú¿144ú⌐/Dreal (13).jpg]

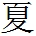

Supplement: S1 Materials — (ZIP) [file pone.0221891.s003.zip › S1_Materials/Drealú¿144ú⌐/Dreal (130).jpg]

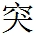

Supplement: S1 Materials — (ZIP) [file pone.0221891.s003.zip › S1_Materials/Drealú¿144ú⌐/Dreal (131).jpg]

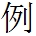

Supplement: S1 Materials — (ZIP) [file pone.0221891.s003.zip › S1_Materials/Drealú¿144ú⌐/Dreal (132).jpg]

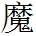

Supplement: S1 Materials — (ZIP) [file pone.0221891.s003.zip › S1_Materials/Drealú¿144ú⌐/Dreal (133).jpg]

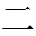

Supplement: S1 Materials — (ZIP) [file pone.0221891.s003.zip › S1_Materials/Drealú¿144ú⌐/Dreal (134).jpg]

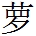

Supplement: S1 Materials — (ZIP) [file pone.0221891.s003.zip › S1_Materials/Drealú¿144ú⌐/Dreal (135).jpg]

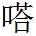

Supplement: S1 Materials — (ZIP) [file pone.0221891.s003.zip › S1_Materials/Drealú¿144ú⌐/Dreal (136).jpg]

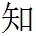

Supplement: S1 Materials — (ZIP) [file pone.0221891.s003.zip › S1_Materials/Drealú¿144ú⌐/Dreal (137).jpg]

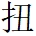

Supplement: S1 Materials — (ZIP) [file pone.0221891.s003.zip › S1_Materials/Drealú¿144ú⌐/Dreal (138).jpg]

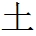

Supplement: S1 Materials — (ZIP) [file pone.0221891.s003.zip › S1_Materials/Drealú¿144ú⌐/Dreal (139).jpg]

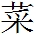

Supplement: S1 Materials — (ZIP) [file pone.0221891.s003.zip › S1_Materials/Drealú¿144ú⌐/Dreal (14).jpg]

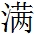

Supplement: S1 Materials — (ZIP) [file pone.0221891.s003.zip › S1_Materials/Drealú¿144ú⌐/Dreal (140).jpg]

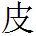

Supplement: S1 Materials — (ZIP) [file pone.0221891.s003.zip › S1_Materials/Drealú¿144ú⌐/Dreal (141).jpg]

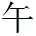

Supplement: S1 Materials — (ZIP) [file pone.0221891.s003.zip › S1_Materials/Drealú¿144ú⌐/Dreal (142).jpg]

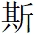

Supplement: S1 Materials — (ZIP) [file pone.0221891.s003.zip › S1_Materials/Drealú¿144ú⌐/Dreal (143).jpg]

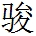

Supplement: S1 Materials — (ZIP) [file pone.0221891.s003.zip › S1_Materials/Drealú¿144ú⌐/Dreal (144).jpg]

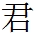

Supplement: S1 Materials — (ZIP) [file pone.0221891.s003.zip › S1_Materials/Drealú¿144ú⌐/Dreal (15).jpg]

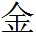

Supplement: S1 Materials — (ZIP) [file pone.0221891.s003.zip › S1_Materials/Drealú¿144ú⌐/Dreal (16).jpg]

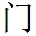

Supplement: S1 Materials — (ZIP) [file pone.0221891.s003.zip › S1_Materials/Drealú¿144ú⌐/Dreal (17).jpg]

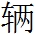

Supplement: S1 Materials — (ZIP) [file pone.0221891.s003.zip › S1_Materials/Drealú¿144ú⌐/Dreal (18).jpg]

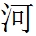

Supplement: S1 Materials — (ZIP) [file pone.0221891.s003.zip › S1_Materials/Drealú¿144ú⌐/Dreal (19).jpg]
