# Supplementary figures and images for: Reading skill modulates the effect of parafoveal distractors on foveal lexical decision in deaf students (part 1 of 2)
Source: PLoS One. 2019 Sep 12;14(9):e0221891. doi: 10.1371/journal.pone.0221891 (PMC6742358; doi:10.1371/journal.pone.0221891)

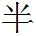

Supplement: S1 Materials — (ZIP) [file pone.0221891.s003.zip › S1_Materials/Dpseudoú¿144ú⌐/Dpseudo (1).jpg]

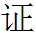

Supplement: S1 Materials — (ZIP) [file pone.0221891.s003.zip › S1_Materials/Dpseudoú¿144ú⌐/Dpseudo (10).jpg]

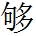

Supplement: S1 Materials — (ZIP) [file pone.0221891.s003.zip › S1_Materials/Dpseudoú¿144ú⌐/Dpseudo (100).jpg]

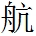

Supplement: S1 Materials — (ZIP) [file pone.0221891.s003.zip › S1_Materials/Dpseudoú¿144ú⌐/Dpseudo (101).jpg]

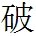

Supplement: S1 Materials — (ZIP) [file pone.0221891.s003.zip › S1_Materials/Dpseudoú¿144ú⌐/Dpseudo (102).jpg]

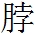

Supplement: S1 Materials — (ZIP) [file pone.0221891.s003.zip › S1_Materials/Dpseudoú¿144ú⌐/Dpseudo (103).jpg]

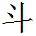

Supplement: S1 Materials — (ZIP) [file pone.0221891.s003.zip › S1_Materials/Dpseudoú¿144ú⌐/Dpseudo (104).jpg]

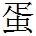

Supplement: S1 Materials — (ZIP) [file pone.0221891.s003.zip › S1_Materials/Dpseudoú¿144ú⌐/Dpseudo (105).jpg]

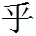

Supplement: S1 Materials — (ZIP) [file pone.0221891.s003.zip › S1_Materials/Dpseudoú¿144ú⌐/Dpseudo (106).jpg]

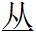

Supplement: S1 Materials — (ZIP) [file pone.0221891.s003.zip › S1_Materials/Dpseudoú¿144ú⌐/Dpseudo (107).jpg]

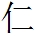

Supplement: S1 Materials — (ZIP) [file pone.0221891.s003.zip › S1_Materials/Dpseudoú¿144ú⌐/Dpseudo (108).jpg]

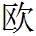

Supplement: S1 Materials — (ZIP) [file pone.0221891.s003.zip › S1_Materials/Dpseudoú¿144ú⌐/Dpseudo (109).jpg]

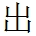

Supplement: S1 Materials — (ZIP) [file pone.0221891.s003.zip › S1_Materials/Dpseudoú¿144ú⌐/Dpseudo (11).jpg]

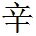

Supplement: S1 Materials — (ZIP) [file pone.0221891.s003.zip › S1_Materials/Dpseudoú¿144ú⌐/Dpseudo (110).jpg]

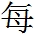

Supplement: S1 Materials — (ZIP) [file pone.0221891.s003.zip › S1_Materials/Dpseudoú¿144ú⌐/Dpseudo (111).jpg]

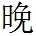

Supplement: S1 Materials — (ZIP) [file pone.0221891.s003.zip › S1_Materials/Dpseudoú¿144ú⌐/Dpseudo (112).jpg]

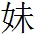

Supplement: S1 Materials — (ZIP) [file pone.0221891.s003.zip › S1_Materials/Dpseudoú¿144ú⌐/Dpseudo (113).jpg]

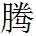

Supplement: S1 Materials — (ZIP) [file pone.0221891.s003.zip › S1_Materials/Dpseudoú¿144ú⌐/Dpseudo (114).jpg]

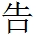

Supplement: S1 Materials — (ZIP) [file pone.0221891.s003.zip › S1_Materials/Dpseudoú¿144ú⌐/Dpseudo (115).jpg]

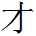

Supplement: S1 Materials — (ZIP) [file pone.0221891.s003.zip › S1_Materials/Dpseudoú¿144ú⌐/Dpseudo (116).jpg]

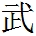

Supplement: S1 Materials — (ZIP) [file pone.0221891.s003.zip › S1_Materials/Dpseudoú¿144ú⌐/Dpseudo (117).jpg]

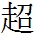

Supplement: S1 Materials — (ZIP) [file pone.0221891.s003.zip › S1_Materials/Dpseudoú¿144ú⌐/Dpseudo (118).jpg]

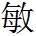

Supplement: S1 Materials — (ZIP) [file pone.0221891.s003.zip › S1_Materials/Dpseudoú¿144ú⌐/Dpseudo (119).jpg]

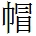

Supplement: S1 Materials — (ZIP) [file pone.0221891.s003.zip › S1_Materials/Dpseudoú¿144ú⌐/Dpseudo (12).jpg]

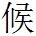

Supplement: S1 Materials — (ZIP) [file pone.0221891.s003.zip › S1_Materials/Dpseudoú¿144ú⌐/Dpseudo (120).jpg]

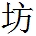

Supplement: S1 Materials — (ZIP) [file pone.0221891.s003.zip › S1_Materials/Dpseudoú¿144ú⌐/Dpseudo (121).jpg]

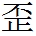

Supplement: S1 Materials — (ZIP) [file pone.0221891.s003.zip › S1_Materials/Dpseudoú¿144ú⌐/Dpseudo (122).jpg]

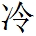

Supplement: S1 Materials — (ZIP) [file pone.0221891.s003.zip › S1_Materials/Dpseudoú¿144ú⌐/Dpseudo (123).jpg]

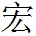

Supplement: S1 Materials — (ZIP) [file pone.0221891.s003.zip › S1_Materials/Dpseudoú¿144ú⌐/Dpseudo (124).jpg]

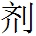

Supplement: S1 Materials — (ZIP) [file pone.0221891.s003.zip › S1_Materials/Dpseudoú¿144ú⌐/Dpseudo (125).jpg]

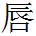

Supplement: S1 Materials — (ZIP) [file pone.0221891.s003.zip › S1_Materials/Dpseudoú¿144ú⌐/Dpseudo (126).jpg]

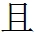

Supplement: S1 Materials — (ZIP) [file pone.0221891.s003.zip › S1_Materials/Dpseudoú¿144ú⌐/Dpseudo (127).jpg]

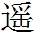

Supplement: S1 Materials — (ZIP) [file pone.0221891.s003.zip › S1_Materials/Dpseudoú¿144ú⌐/Dpseudo (128).jpg]

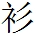

Supplement: S1 Materials — (ZIP) [file pone.0221891.s003.zip › S1_Materials/Dpseudoú¿144ú⌐/Dpseudo (129).jpg]

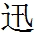

Supplement: S1 Materials — (ZIP) [file pone.0221891.s003.zip › S1_Materials/Dpseudoú¿144ú⌐/Dpseudo (13).jpg]

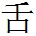

Supplement: S1 Materials — (ZIP) [file pone.0221891.s003.zip › S1_Materials/Dpseudoú¿144ú⌐/Dpseudo (130).jpg]

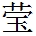

Supplement: S1 Materials — (ZIP) [file pone.0221891.s003.zip › S1_Materials/Dpseudoú¿144ú⌐/Dpseudo (131).jpg]

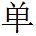

Supplement: S1 Materials — (ZIP) [file pone.0221891.s003.zip › S1_Materials/Dpseudoú¿144ú⌐/Dpseudo (132).jpg]

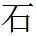

Supplement: S1 Materials — (ZIP) [file pone.0221891.s003.zip › S1_Materials/Dpseudoú¿144ú⌐/Dpseudo (133).jpg]

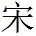

Supplement: S1 Materials — (ZIP) [file pone.0221891.s003.zip › S1_Materials/Dpseudoú¿144ú⌐/Dpseudo (134).jpg]

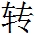

Supplement: S1 Materials — (ZIP) [file pone.0221891.s003.zip › S1_Materials/Dpseudoú¿144ú⌐/Dpseudo (135).jpg]

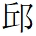

Supplement: S1 Materials — (ZIP) [file pone.0221891.s003.zip › S1_Materials/Dpseudoú¿144ú⌐/Dpseudo (136).jpg]

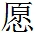

Supplement: S1 Materials — (ZIP) [file pone.0221891.s003.zip › S1_Materials/Dpseudoú¿144ú⌐/Dpseudo (137).jpg]

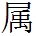

Supplement: S1 Materials — (ZIP) [file pone.0221891.s003.zip › S1_Materials/Dpseudoú¿144ú⌐/Dpseudo (138).jpg]

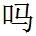

Supplement: S1 Materials — (ZIP) [file pone.0221891.s003.zip › S1_Materials/Dpseudoú¿144ú⌐/Dpseudo (139).jpg]

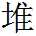

Supplement: S1 Materials — (ZIP) [file pone.0221891.s003.zip › S1_Materials/Dpseudoú¿144ú⌐/Dpseudo (14).jpg]

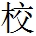

Supplement: S1 Materials — (ZIP) [file pone.0221891.s003.zip › S1_Materials/Dpseudoú¿144ú⌐/Dpseudo (140).jpg]

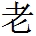

Supplement: S1 Materials — (ZIP) [file pone.0221891.s003.zip › S1_Materials/Dpseudoú¿144ú⌐/Dpseudo (141).jpg]

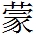

Supplement: S1 Materials — (ZIP) [file pone.0221891.s003.zip › S1_Materials/Dpseudoú¿144ú⌐/Dpseudo (142).jpg]

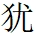

Supplement: S1 Materials — (ZIP) [file pone.0221891.s003.zip › S1_Materials/Dpseudoú¿144ú⌐/Dpseudo (143).jpg]

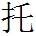

Supplement: S1 Materials — (ZIP) [file pone.0221891.s003.zip › S1_Materials/Dpseudoú¿144ú⌐/Dpseudo (144).jpg]

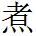

Supplement: S1 Materials — (ZIP) [file pone.0221891.s003.zip › S1_Materials/Dpseudoú¿144ú⌐/Dpseudo (15).jpg]

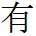

Supplement: S1 Materials — (ZIP) [file pone.0221891.s003.zip › S1_Materials/Dpseudoú¿144ú⌐/Dpseudo (16).jpg]

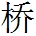

Supplement: S1 Materials — (ZIP) [file pone.0221891.s003.zip › S1_Materials/Dpseudoú¿144ú⌐/Dpseudo (17).jpg]

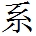

Supplement: S1 Materials — (ZIP) [file pone.0221891.s003.zip › S1_Materials/Dpseudoú¿144ú⌐/Dpseudo (18).jpg]

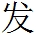

Supplement: S1 Materials — (ZIP) [file pone.0221891.s003.zip › S1_Materials/Dpseudoú¿144ú⌐/Dpseudo (19).jpg]

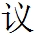

Supplement: S1 Materials — (ZIP) [file pone.0221891.s003.zip › S1_Materials/Dpseudoú¿144ú⌐/Dpseudo (2).jpg]

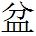

Supplement: S1 Materials — (ZIP) [file pone.0221891.s003.zip › S1_Materials/Dpseudoú¿144ú⌐/Dpseudo (20).jpg]

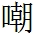

Supplement: S1 Materials — (ZIP) [file pone.0221891.s003.zip › S1_Materials/Dpseudoú¿144ú⌐/Dpseudo (21).jpg]

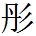

Supplement: S1 Materials — (ZIP) [file pone.0221891.s003.zip › S1_Materials/Dpseudoú¿144ú⌐/Dpseudo (22).jpg]

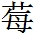

Supplement: S1 Materials — (ZIP) [file pone.0221891.s003.zip › S1_Materials/Dpseudoú¿144ú⌐/Dpseudo (23).jpg]

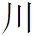

Supplement: S1 Materials — (ZIP) [file pone.0221891.s003.zip › S1_Materials/Dpseudoú¿144ú⌐/Dpseudo (24).jpg]

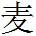

Supplement: S1 Materials — (ZIP) [file pone.0221891.s003.zip › S1_Materials/Dpseudoú¿144ú⌐/Dpseudo (25).jpg]

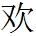

Supplement: S1 Materials — (ZIP) [file pone.0221891.s003.zip › S1_Materials/Dpseudoú¿144ú⌐/Dpseudo (26).jpg]

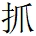

Supplement: S1 Materials — (ZIP) [file pone.0221891.s003.zip › S1_Materials/Dpseudoú¿144ú⌐/Dpseudo (27).jpg]

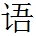

Supplement: S1 Materials — (ZIP) [file pone.0221891.s003.zip › S1_Materials/Dpseudoú¿144ú⌐/Dpseudo (28).jpg]

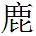

Supplement: S1 Materials — (ZIP) [file pone.0221891.s003.zip › S1_Materials/Dpseudoú¿144ú⌐/Dpseudo (29).jpg]

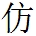

Supplement: S1 Materials — (ZIP) [file pone.0221891.s003.zip › S1_Materials/Dpseudoú¿144ú⌐/Dpseudo (3).jpg]

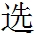

Supplement: S1 Materials — (ZIP) [file pone.0221891.s003.zip › S1_Materials/Dpseudoú¿144ú⌐/Dpseudo (30).jpg]

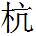

Supplement: S1 Materials — (ZIP) [file pone.0221891.s003.zip › S1_Materials/Dpseudoú¿144ú⌐/Dpseudo (31).jpg]

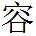

Supplement: S1 Materials — (ZIP) [file pone.0221891.s003.zip › S1_Materials/Dpseudoú¿144ú⌐/Dpseudo (32).jpg]

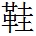

Supplement: S1 Materials — (ZIP) [file pone.0221891.s003.zip › S1_Materials/Dpseudoú¿144ú⌐/Dpseudo (33).jpg]

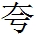

Supplement: S1 Materials — (ZIP) [file pone.0221891.s003.zip › S1_Materials/Dpseudoú¿144ú⌐/Dpseudo (34).jpg]

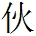

Supplement: S1 Materials — (ZIP) [file pone.0221891.s003.zip › S1_Materials/Dpseudoú¿144ú⌐/Dpseudo (35).jpg]

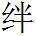

Supplement: S1 Materials — (ZIP) [file pone.0221891.s003.zip › S1_Materials/Dpseudoú¿144ú⌐/Dpseudo (36).jpg]

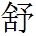

Supplement: S1 Materials — (ZIP) [file pone.0221891.s003.zip › S1_Materials/Dpseudoú¿144ú⌐/Dpseudo (37).jpg]

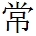

Supplement: S1 Materials — (ZIP) [file pone.0221891.s003.zip › S1_Materials/Dpseudoú¿144ú⌐/Dpseudo (38).jpg]

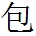

Supplement: S1 Materials — (ZIP) [file pone.0221891.s003.zip › S1_Materials/Dpseudoú¿144ú⌐/Dpseudo (39).jpg]

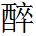

Supplement: S1 Materials — (ZIP) [file pone.0221891.s003.zip › S1_Materials/Dpseudoú¿144ú⌐/Dpseudo (4).jpg]

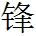

Supplement: S1 Materials — (ZIP) [file pone.0221891.s003.zip › S1_Materials/Dpseudoú¿144ú⌐/Dpseudo (40).jpg]

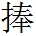

Supplement: S1 Materials — (ZIP) [file pone.0221891.s003.zip › S1_Materials/Dpseudoú¿144ú⌐/Dpseudo (41).jpg]

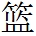

Supplement: S1 Materials — (ZIP) [file pone.0221891.s003.zip › S1_Materials/Dpseudoú¿144ú⌐/Dpseudo (42).jpg]

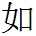

Supplement: S1 Materials — (ZIP) [file pone.0221891.s003.zip › S1_Materials/Dpseudoú¿144ú⌐/Dpseudo (43).jpg]

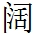

Supplement: S1 Materials — (ZIP) [file pone.0221891.s003.zip › S1_Materials/Dpseudoú¿144ú⌐/Dpseudo (44).jpg]

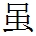

Supplement: S1 Materials — (ZIP) [file pone.0221891.s003.zip › S1_Materials/Dpseudoú¿144ú⌐/Dpseudo (45).jpg]

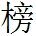

Supplement: S1 Materials — (ZIP) [file pone.0221891.s003.zip › S1_Materials/Dpseudoú¿144ú⌐/Dpseudo (46).jpg]

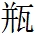

Supplement: S1 Materials — (ZIP) [file pone.0221891.s003.zip › S1_Materials/Dpseudoú¿144ú⌐/Dpseudo (47).jpg]

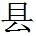

Supplement: S1 Materials — (ZIP) [file pone.0221891.s003.zip › S1_Materials/Dpseudoú¿144ú⌐/Dpseudo (48).jpg]

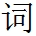

Supplement: S1 Materials — (ZIP) [file pone.0221891.s003.zip › S1_Materials/Dpseudoú¿144ú⌐/Dpseudo (49).jpg]

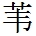

Supplement: S1 Materials — (ZIP) [file pone.0221891.s003.zip › S1_Materials/Dpseudoú¿144ú⌐/Dpseudo (5).jpg]

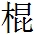

Supplement: S1 Materials — (ZIP) [file pone.0221891.s003.zip › S1_Materials/Dpseudoú¿144ú⌐/Dpseudo (50).jpg]

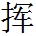

Supplement: S1 Materials — (ZIP) [file pone.0221891.s003.zip › S1_Materials/Dpseudoú¿144ú⌐/Dpseudo (51).jpg]

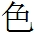

Supplement: S1 Materials — (ZIP) [file pone.0221891.s003.zip › S1_Materials/Dpseudoú¿144ú⌐/Dpseudo (52).jpg]

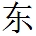

Supplement: S1 Materials — (ZIP) [file pone.0221891.s003.zip › S1_Materials/Dpseudoú¿144ú⌐/Dpseudo (53).jpg]

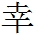

Supplement: S1 Materials — (ZIP) [file pone.0221891.s003.zip › S1_Materials/Dpseudoú¿144ú⌐/Dpseudo (54).jpg]

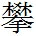

Supplement: S1 Materials — (ZIP) [file pone.0221891.s003.zip › S1_Materials/Dpseudoú¿144ú⌐/Dpseudo (55).jpg]

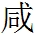

Supplement: S1 Materials — (ZIP) [file pone.0221891.s003.zip › S1_Materials/Dpseudoú¿144ú⌐/Dpseudo (56).jpg]

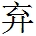

Supplement: S1 Materials — (ZIP) [file pone.0221891.s003.zip › S1_Materials/Dpseudoú¿144ú⌐/Dpseudo (57).jpg]

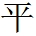

Supplement: S1 Materials — (ZIP) [file pone.0221891.s003.zip › S1_Materials/Dpseudoú¿144ú⌐/Dpseudo (58).jpg]

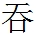

Supplement: S1 Materials — (ZIP) [file pone.0221891.s003.zip › S1_Materials/Dpseudoú¿144ú⌐/Dpseudo (59).jpg]
